# Excellent Outcome Following Sibling Peripheral Blood Hematopoietic Stem Cell Transplantation for Glanzmann Thrombasthenia: A Case Report

**DOI:** 10.3389/fped.2021.776927

**Published:** 2022-02-07

**Authors:** Jian hua Li, Shu wen Sun, Yuan Ai, Xue Yang, Yi ping Zhu

**Affiliations:** ^1^Department of Pediatrics, West China Second University Hospital, Sichuan University, Chengdu, China; ^2^Key Laboratory of Birth Defects and Related Diseases of Women and Children, Sichuan University, Ministry of Education, Chengdu, China

**Keywords:** bone marrow transplantation, peripheral blood stem cell transplantation, platelet disorder, Glanzmann thrombasthenia, sibling

## Abstract

Glanzmann thrombasthenia (GT) is a rare autosomal recessive platelet disorder due to a qualitative or quantitative anomaly of the platelet membrane glycoprotein GPIIb/IIIa. Its clinical manifestations include mild to severe bleeding. GT diagnosis mainly depends on platelet function analysis, flow cytometry, and gene detection. Treatment methods include conservative symptomatic treatment and allogeneic hematopoietic stem cell transplantation (allo-HSCT). Allo-HSCT is the only clinical radical method for GT. Herein, we report a 2-year-old boy with GT successfully cured by related identical peripheral blood stem cell transplantation (PBSCT). The platelet disorder was corrected to a normal level after PBSCT, with no significant complication related to the transplantation. Hematopoietic stem cell transplantation with full-matched donor in early stage could be a treatment option for GT.

## Introduction

Glanzmann thrombasthenia (GT) is a rare inherited platelet disorder, occurring in an autosomal recessive manner. Its incidence rate is 1/million, which increases to 1/200,000 in areas of high consanguinity (1). Patients often experience purpura, epistaxis, gingival hemorrhage, and abnormal bleeding after trauma or surgery. Mutations of the ITGA2B or ITGB3 gene on chromosome 17 cause deficits or reduced expression of the platelet membrane glycoprotein GPIIb/IIIa, an important fibrinogen receptor (2). The binding of fibrinogen to GPIIb/IIIa causes platelet aggregation, forming a thrombus and participating in the hemostasis process. Patients with GPIIb/IIIa defect often experience mild to severe bleeding, and even life-threatening bleeding events. Epistaxis is the main cause of severe bleeding (3). Studies revealed prolonged bleeding time, normal platelet count and morphology, and normal coagulation. GT diagnosis is mainly based on absent or severely diminished platelet aggregation by multiple platelet agonists such as adenosine diphosphate, arachidonic acid, adrenaline, collagen, and thrombin. Flow cytometry examination and genetic testing are used to confirm the diagnosis (4). Antifibrinolytic drugs are effective for mild bleeding, and desmopressin has little effects. Platelet transfusion is the most effective treatment for hemostasis, but recurrent platelet transfusion promotes the production of anti-HLA and anti-GP IIb/IIIa antibodies. The platelet antibody production rate in GT patients is as high as 30% (5).

Infusion of HLA-matched platelets prevents the production of HLA antibodies. However, there is currently no method to predict or avoid the production of GPIIb/IIIa antibodies. In patients with platelet resistance, recombinant human activated factor VII (rVIIa) along with high dose of platelets should be administered. Allogeneic hematopoietic stem cell transplantation (allo-HSCT) is the only definitive therapeutic method for GT. Here, we adopt sibling peripheral blood stem cell transplantation to treat GT.

## Case Report

**Figure 1:**
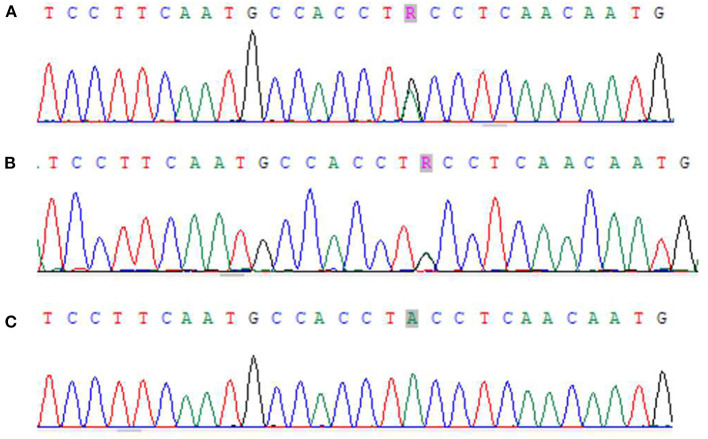
Next-generation sequencing results of zhou xx and his parents. **(A)** Zhou xx - Father ITGB3 exon9:c.1199G>A showed G/A heterozygosity. **(B)** Zhou xx - mather ITGB3 exon9:c.1199G>A showed G/A heterozygosity. **(C)** Zhou xx - ITGB3 exon9:c.1199G>A showed G/A homozygosity.

**Figure 2:**
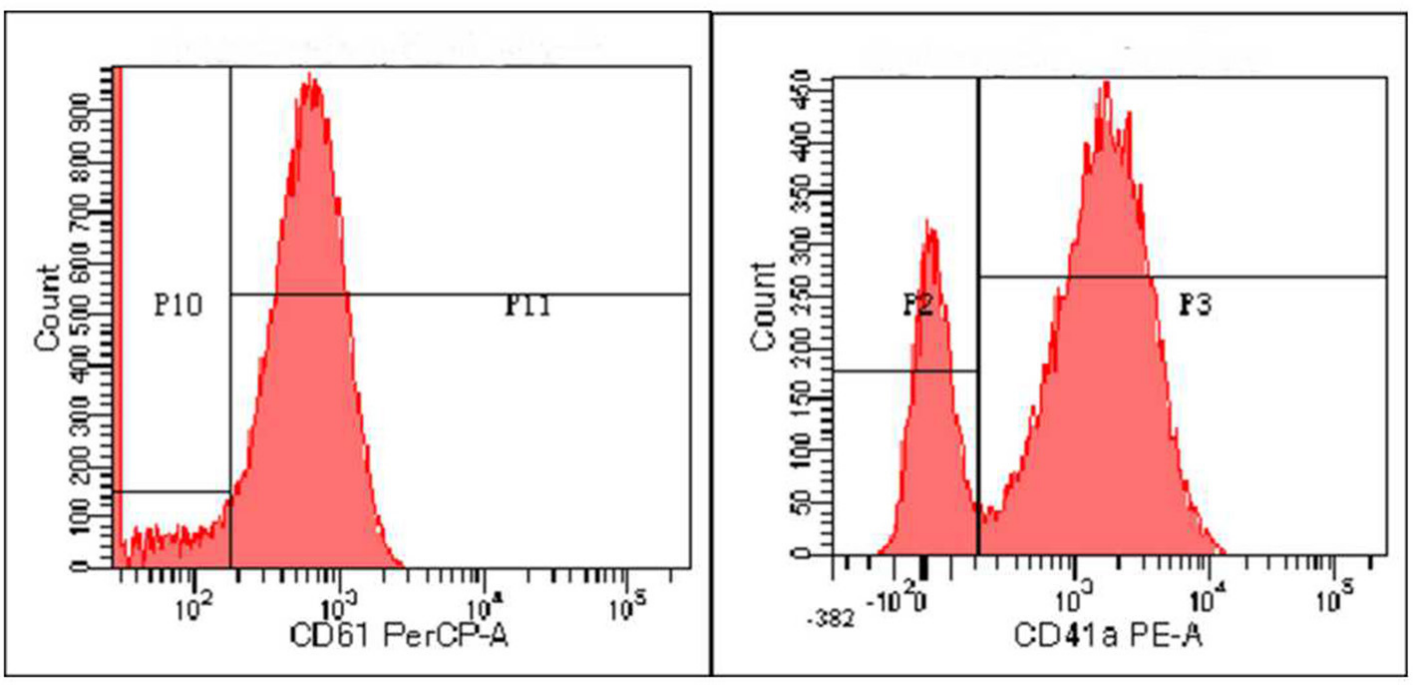
The expression of CD61 and CD41a after transplantation detected by flow cytometry.

Zhou XX, male, 32 months old, suffered from recurrent mucocutaneous bleeding after birth. His parents had a consanguineous marriage. A sister and a brother both died at the age of three from severe bleeding caused by epistaxis. He was admitted to West China Second Hospital of Sichuan University due to recurrent epistaxis at the age of 29 months old. The epistaxis occurred once a month and lasted for about 3–8 days, and local therapeutic and hemostatic drugs were ineffective; transfusion of platelets and red blood cells was needed. He received four platelet and one red cell transfusions. There was no fever, joint swelling, hepatomegaly, or splenomegaly during the disease. Laboratory examination showed normal platelet count and morphology, as well as normal blood coagulation function. Therefore, immune thrombocytopenia, hemophilia, and leukemia were ruled out. Inherited platelet dysfunction should be considered in infants with recurrent bleeding disorders if platelet count, morphology, and coagulation function are normal. Consanguineous marriage and siblings dead of unexplained hemorrhagic are supportive of the diagnosis (6). In addition, flow cytometry examination in a local hospital suggested decreased expression of platelet membrane glycoprotein. The proportion of CD61 (GP IIIa) was 32.4%, and CD41a (GP IIb/IIIa) rate was 56.2%. Therefore, we performed a platelet function assay, and found abnormal platelet aggregation, with a maximum ADP aggregation rate of 16.6% (normal 50–100%) and an AA of 29.9% (normal 60–100%). Genetic examination revealed that the child had a homozygous mutation at ITGB3 (ITGB3:exon9:c.1199G>A), and heterozygous mutations were also found in his parents, as shown in Figure 1. Due to the young age at onset, gene sequencing suggested ITGB3 mutation, and acquired Glanzmann thrombasthenia was not considered. The patient was diagnosed with GT (Type III, with >20% expression of platelet membrane glycoprotein, but defective receptor function with variable platelet agonists and clot retraction) (5). Since his elder sister, 9 years old, was a heterozygous carrier with no bleeding manifestation and an identical HLA matched donor, his parents requested allogeneic hematopoietic stem cell transplantation for cure. Therefore, HLA-matched sibling transplantation was performed on March 13, 2020. He was administered myeloablative conditioning (busulfan 20 mg/kg for 4 days and cyclophosphamide 200 mg/kg for 4 days) and received 29 ml of peripheral blood stem cells from his HLA identical sister, containing CD34+ cells at 6.0^*^10^∧^6/kg, TNCs at 3.4^*^10^∧^8/kg, MNCs at 7.6^*^10^∧^8/kg, CD3+ cells at 2.2^*^10^∧^8/kg, and B lymphocytes at 4.5^*^10^∧^6/kg. Cyclosporine (CSA) and a short course of methotrexate were administered for graft-vs.-host disease (GVHD) prophylaxis. Neutrophils and platelets were engrafted at +11 days post-transplantation. Chimerism test indicated complete donor type at +20 days post-transplantation. Flow cytometry examination confirmed the proportion of CD61 (GP IIIa) was 84.6%, and CD41a (GP IIb/IIIa) rate was 85.3% on the 25th day after PBSCT (Figure 2). CD61 and CD41a expression rates were significantly higher than those observed before transplantation (Supplementary Table 1). One month after transplantation, the patient developed Epstein-Barr viremia with 6.35^*^10^∧^5 copies of EBV DNA/ml, which was cured after 2 weeks of acyclovir antiviral therapy. The child did not develop GVHD and tapered CSA after 6 months of transplantation. There were no complications and no bleeding events at 20 months post-transplantation.

## Discussion

We here report a rare case of GT Type III case with successful matched sibling donor transplantation using myeloablative conditioning. Hematopoietic stem cell transplantation for the treatment of GT has long been established. However, there are no large-sample studies assessing HSCT in GT patients, and its indications are unclear. Considering its complications, HSCT is mainly recommended for GT with recurring life-threatening bleeding or platelet resistance. For the current patient, transplantation decision was justified by the early mortality of two siblings and the optimal hematopoietic stem cell source. In addition, considering cost and compliance, our patient could benefit more from transplantation than from conservative treatment. Therefore, in order to reduce the risk of transfusion exposure and homogeneous immune response, and to decrease the odds of transplant-related organ dysfunction, we performed transplantation in the early stage.

Acquisition of hematopoietic stem cells and complications are the main reasons preventing HSCT for GT. Due to the difficulty in finding a suitable donor, cord blood stem cell transfusion is considered. However, it is associated with slow hematopoietic recovery and high incidence of acute GVHD (7–9). Although peripheral hematopoietic stem cells selected in this work may increase the risk of chronic GVHD, a full-matched sibling donor was selected, which could reduce the risk of acute or chronic GVHD compared with alternative donors. Indeed, this has also been confirmed. Furthermore, we used myeloablative conditioning (MAC), which results in faster implantation, better reconstruction of the hematopoietic system, and lower transplant-related infections, compared with reduced intensity conditioning (RIC). Walz et al. (10) reported an 8-month-old GT patient who underwent a successful matched sibling donor transplantation using reduced intensity conditioning, and neutrophils and platelets were implanted at +18 post-transplantation, later than in the current patient (+11 post-transplantation). Connor et al. (11) reported a case of GT who underwent reduced intensity conditioning, and the chimerism rate was only 30%. In WAS syndrome, a large-scale retrospective analysis showed that MAC achieves better donor chimerism in the early post-transplantation period compared with RIC (12). Therefore, reduced intensity conditioning decreases chemotherapy-drug toxicity but also increases the risk of rejection and poor implantation.

The type of gene mutation and GPIIb/IIIa expression rate are not associated with the severity of GT (13). There is no effective indicator to predict the severity of bleeding in an individual. HLA identical sibling peripheral blood stem cell transplantation for GT is safe and effective. Using myeloablative conditioning and early transplant could reduce the incidence of transplantation related complications, prolong the patient's life, and improve prognosis.

## Data Availability Statement

The original contributions presented in the study are included in the article/Supplementary Material, further inquiries can be directed to the corresponding author/s.

## Ethics Statement

Ethical review and approval was not required for the study on human participants in accordance with the local legislation and institutional requirements. Written informed consent to participate in this study was provided by the participants' legal guardian/next of kin.

## Author Contributions

JL and SS carried out the studies, participated in data collection, and drafted the manuscript. XY and YA performed the statistical analysis and participated in study design. XY and YZ participated in the acquisition, analysis, interpretation of data, and drafted the manuscript. All authors have read and approved the final manuscript.

## Author Disclaimer

The views expressed in this article are solely those of the authors and do not necessarily represent those of their affiliated organizations, or those of the publisher, the editors, and the reviewers. Any product that may be evaluated in this article or claim that may be made by its manufacturer is not guaranteed or endorsed by the publisher.

## Conflict of Interest

The authors declare that the research was conducted in the absence of any commercial or financial relationships that could be construed as a potential conflict of interest.

## Publisher's Note

All claims expressed in this article are solely those of the authors and do not necessarily represent those of their affiliated organizations, or those of the publisher, the editors and the reviewers. Any product that may be evaluated in this article, or claim that may be made by its manufacturer, is not guaranteed or endorsed by the publisher.
