# Supplementary figures and images for: Excellent Outcome Following Sibling Peripheral Blood Hematopoietic Stem Cell Transplantation for Glanzmann Thrombasthenia: A Case Report
Source: Front Pediatr. 2022 Feb 7;9:776927. doi: 10.3389/fped.2021.776927 (PMC8859262; doi:10.3389/fped.2021.776927)

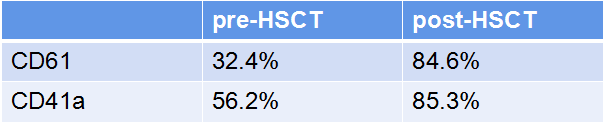


Table 1

Expression rate of platelet membrane glycoprotein before and after transplantation

Supplement: Supplementary file 1 [file Table_1.docx]
